# Maternal Obesity During Pregnancy and Lactation Influences Offspring Obesogenic Adipogenesis but Not Developmental Adipogenesis in Mice

**DOI:** 10.3390/nu11030495

**Published:** 2019-02-27

**Authors:** Dyan Sellayah, Hugh Thomas, Stuart A. Lanham, Felino R. Cagampang

**Affiliations:** 1 Harborne Building 12A, School of Biological Sciences, University of Reading, Whiteknights, Reading, Berkshire RG6 6AS, UK; 2 Institute of Developmental Sciences, School of Medicine, University of Southampton, Southampton General Hospital, Tremona Road, Southampton SO16 6YD, UK; hugh.thomas@nature.com (H.T.); s.a.lanham@soton.ac.uk (S.A.L.); f.cagampang@soton.ac.uk (F.R.C.)

**Keywords:** maternal obesity, nutrition, programming

## Abstract

Obesity is an escalating health crisis of pandemic proportions and by all accounts it has yet to reach its peak. Growing evidence suggests that obesity may have its origins in utero. Recent studies have shown that maternal obesity during pregnancy may promote adipogenesis in offspring. However, these studies were largely based on cell culture models. Whether or not maternal obesity impacts on offspring adipogenesis in vivo remains to be fully established. Furthermore, in vivo adipogenic differentiation has been shown to happen at distinct time periods, one during development (developmental adipogenesis—which is complete by 4 weeks of age in mice) and another in adulthood in response to feeding a high-fat (HF) diet (obesogenic adipogenesis). We therefore set out to determine whether maternal obesity impacted on offspring adipocyte hyperplasia in vivo and whether maternal obesity impacted on developmental or obesogenic adipogenesis, or both. Our findings reveal that maternal obesity is associated with enhanced obesogenic adipogenesis in HF-fed offspring. Interestingly, in newly weaned (4-week-old) offspring, maternal obesity is associated with adipocyte hypertrophy, but there were no changes in adipocyte number. Our results suggest that maternal obesity impacts on offspring obesogenic adipogenesis but does not affect developmental adipogenesis.

## 1. Introduction

Obesity is an alarming threat of pandemic proportions. Its economic impact is crippling, costing the global economy tens of billions of U.S. dollars each year [1]. An inevitable consequence of the upsurge in obesity rates in the general population is an increased prevalence of obesity during pregnancy [2]. In addition to this, recent years have seen an alarming rise in excessive nutrient intake during gestation in developed countries [3]. These trends are particularly concerning in light of numerous animal models showing that maternal obesity and high-fat (HF) or high-calorie consumption during pregnancy predisposes offspring to obesity and metabolic disease in later life [4,5,6]. How maternal obesity and overnutrition during pregnancy promotes obesity in offspring remains to be fully determined. One potential mechanism underlying the causal relationship between maternal obesity during pregnancy and increased offspring adiposity and obesity susceptibility in later life is the programming of adipogenesis in offspring white adipose tissue (WAT). 

Adipogenesis is the process by which adipose tissue expands through hyperplastic growth [7]. As mature adipocytes are post-mitotic, adipogenesis must rely on de novo adipocyte differentiation from adipocyte progenitors (APs) [8]. Adipose tissue exists in various forms with diverse functions and is widely distributed in distinct locations throughout the mammalian body. Whilst brown adipose tissue and subcutaneous white adipose tissue have beneficial effects on metabolic health [9,10], visceral adipose tissue has been shown to be deleterious. Visceral adipose tissue expansion in obesity is strongly associated with inflammation, leading to metabolic dysfunction including cardiovascular disease and insulin resistance [11,12]. Much effort in recent years has been dedicated to understanding the mechanisms that give rise to adipogenesis in visceral WAT using rodent models. 

In recent years, several distinct phases of adipogenesis of visceral WAT have been identified: developmental adipogenesis, which is responsible for initial tissue formation during development (organogenesis); homeostatic adipogenesis, which is concerned with maintenance/basal turnover of the tissue; and adipose tissue expansion in response to external stimuli such as high-fat diet (obesogenic adipogenesis) or cold exposure [12]. Developmental adipogenesis and adulthood adipogenesis were long thought to be governed by the same regulatory mechanisms, however, in recent years this view has been sharply changed. Adult and developmental adipogenesis have been shown to be regulated by distinct transcriptional [13] and signalling mechanisms [14], and to require different subpopulations of APs [15,16]. 

Recent rodent studies have revealed increased adipogenic capacity of mouse embryonic fibroblasts as well as gonadal white adipose tissue (gWAT)-derived stromal vascular fraction (SVF) cells of offspring of obese mothers (i.e., dams) [17,18]. Furthermore, a recent study in humans has documented enhanced adipogenic capacity of umbilical cord stem cells of infants born to obese dams [19]. While these studies suggest that maternal obesity may induce adipogenesis in offspring, important experimental details are yet to be confirmed. The aforementioned studies documenting increased adipogenic capacity in offspring in response to maternal obesity represent ex vivo studies. Whether this increased adipogenic capacity translates into in vivo effects (i.e., increased adipocyte number is the gWAT) in offspring has not been established. Moreover, whether maternal obesity affects offspring developmental adipogenesis or whether it influences offspring obesogenic adipogenesis in adulthood remains to be fully explored. This is particularly important in light of emerging evidence showing that the various adipogenic processes are controlled by distinct regulatory mechanisms and signalling pathways, while also utilizing different subpopulations of APs.

We therefore established a mouse model of maternal high-fat (HF) diet-induced obesity during pregnancy and lactation, coupled with offspring HF diet-induced obesity. We analysed adipocyte hypertrophic and hyperplastic response through the histological assessment of gWAT of offspring at weanling and at 26 weeks post-weaning.

## 2. Methods

### 2.1. Animal Experiments

All animal procedures were conducted in accordance with the United Kingdom Animals (Scientific Procedures) Act 1986 and were approved by the Faculty of Medicine ethics review committee. Female C57/BL6J mice were maintained under a 12 h light–dark cycle (lights switched on daily at 07.00 h) and maintained at a constant temperature of 22 ± 2 °C with food and water available ad libitum. Dams were randomly assigned to one of two dietary regimes: control (Chow (C); 7% kcal fat; Special Dietary Services Ltd., Witham, UK), or a high-fat diet (HF; 45% kcal fat; Special Dietary Services Ltd., Witham, UK). The nutritional composition of the two diets is shown in Table 1. This HF diet has previously been used to induce adiposity and weight gain in both pregnant dams and their offspring [20]. Dams were fed the designated diet for 6 weeks pre-pregnancy (HF-fed female mice were on average 10 g heavier than chow-fed females prior to mating). Pregnant dams were continuously fed the same pre-pregnancy diet through pregnancy and lactation. Once the pregnant dams delivered their pups, litter size was standardized to six pups (three males and three females, wherever possible). Offspring from each maternal nutritional paradigm were randomly assigned to either the C or HF diet at weaning (4 weeks of age) resulting in four offspring groups: C/C, C/HF, HF/C, and HF/HF—letters before forward slash denote maternal diet, while letters after forward slash denote offspring diet post-weaning (i.e., maternal/offspring diet). Sample sizes were 5–7 per offspring group. Offspring were fed the diets for the next 26 weeks. From 29 weeks of age, the oestrous cyclicity (assessed by vaginal smear) was monitored in the female offspring. At 30 weeks of age both male and female offspring were weighed and then killed by cervical dislocation. Female offspring were killed at dioestrus. The gonadal adipose tissue (the largest visceral adipose tissue depot in these mice) was weighed and either immediately snap frozen in liquid nitrogen and stored at −80 °C or fixed and paraffin embedded for histological analysis. A subset of mice were sacrificed at 4 weeks of age, which is when developmental adipogenesis of gWAT is complete [21].

### 2.2. Assessment of Total Adiposity in 30-Week-Old Offspring

Total adiposity of the offspring was assessed after they were killed at 30 weeks of age by scanning the whole animal using a Skyscan 1176 in vivo micro-CT scanner (Bruker microCT, Kontich, Belgium) using a current of 500 µA, voltage of 50 kV, and with a 0.5-mm aluminium filter. Images were captured at 0.1° intervals of rotation over a 180° rotation. NRecon software (Skyscan, Kontich, Belgium) was used to reconstruct individual images into a 3D model. VGStudio Max version 1.2.1 was used to analyse the reconstructed volume. The x-ray absorbance of the volume produced three peaks which corresponded to bone, fat, and soft tissue. The total volume of each of these component tissue types was measured by setting the thresholding limits to contain the relevant absorbance peak (grey scale 11–20 (fat tissue), 21–40 (soft tissue), and 41–255 (bone)). 

### 2.3. Gene Expression

Gene expression was determined by real-time quantitative PCR utilizing the TaqMan assay system (probes and reagents purchased from Fisher Scientific, Loughborough, UK). Assays were run on an ABI Prism 7700 Sequence Detection machine (Perkin Elmer). Genes examined were *Cd34*, *Zf423*, and *Fto*, which are highly expressed in committed adipocyte progenitor cells. In the case of *Cd34*, expression is absent in mature adipocytes [8]. Samples were measured in duplicate, and target gene expression was normalized to the housekeeping/reference gene glyceraldehyde-3-phosphate dehydrogenase (*Gapdh*). 

### 2.4. Histology

Serial sections of formalin-fixed gWAT of 30-week-old female mouse offspring were stained with haematoxylin and eosin (H&E) for visualization of basic cell morphology. Image-J was used to measure mean adipocyte volume per depot. Adipocyte number per depot was calculated from volume estimates using the equation as we have previously described [22]:

*n* = *m* (depot mass g)/*P* (density of adipose 0.915 g cm^−3^) × volume (cm^3^) (1)

## 3. Results

### 3.1. Maternal Obesity Leads to Increased Body Weight and Adiposity in Offspring Which is Exacerbated by a Post-Weaning HF-Diet

**Figure 1:**
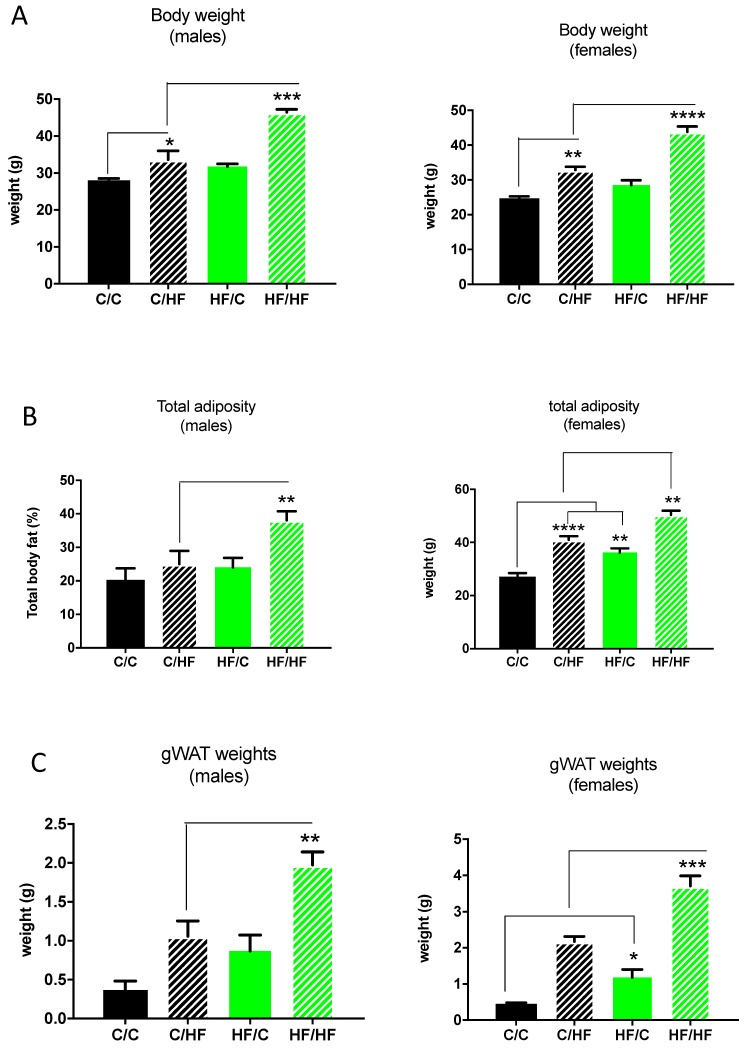
Body weight (**A**), total adiposity (**B**), and gonadal white adipose tissue (gWAT) weights (**C**) in male and female chow (C)- or high-fat (HF)-fed 30-week-old offspring of dams fed a chow C or HF diet for 6 weeks prior to pregnancy and during pregnancy and lactation. All data represent means ± SEM. Data was analysed by ANOVA and Tukey’s multiple comparison test. *N* = 5–7. * *p* < 0.05, ** *p* < 0.01, *** *p* < 0.001, **** *p* < 0.0001.

There were no significant differences in body weight in male and female chow-fed offspring of obese dams compared to chow-fed offspring of lean dams (HF/C vs. C/C (Figure 1A). Both male and female HF-fed offspring of obese dams exhibited increased body weight compared with HF-fed offspring of lean dams (C/HF vs. HF/HF), suggesting a maternal–offspring dietary interaction to bring about changes in body weight (Figure 1A). The HF-diet induced weight gain in offspring of obese dams was considerably greater than in offspring of lean dams (Figure 1A). In male offspring, maternal obesity alone was not associated with changes in total adiposity (HF/C vs. C/C) (Figure 1B). On the other hand, female chow-fed offspring of obese dams had a significantly higher total adiposity compared with chow-fed offspring of lean dams (C/C vs. HF/C) (Figure 1B). HF-diet feeding in male and female offspring of obese dams led to an increased total adiposity compared to HF feeding in offspring of lean dams (C/HF vs. HF/HF) (Figure 1B). Total adiposity measurements were reflected in gonadal white adipose tissue (gWAT) weights wherein male and female chow-fed offspring of obese dams (HF/C) had increased gWAT weights (reaching statistical significance in females only). Post-weaning HF-diet feeding in offspring of obese dams greatly increased gWAT weight compared with HF-fed offspring of lean dams in both males and females (C/HF vs. FH/HF) (Figure 1C). 

### 3.2. Maternal Obesity Impacts on Adipocyte Volume and Number

**Figure 2:**
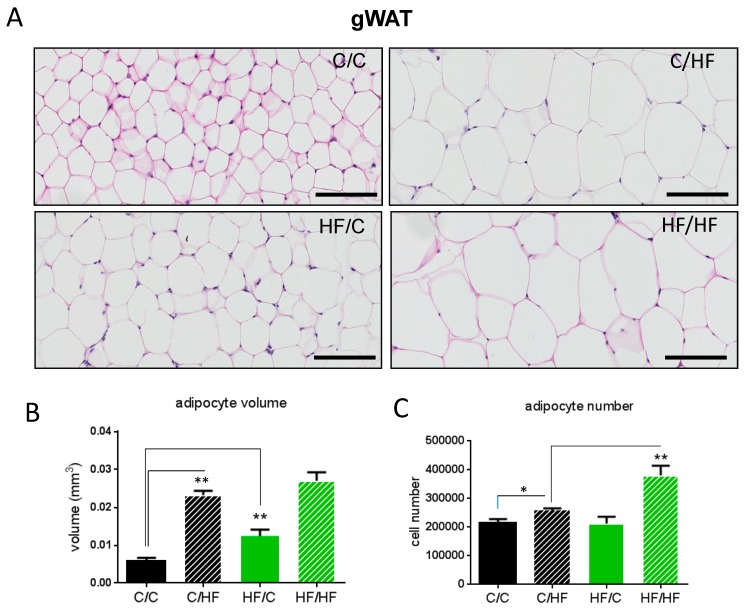
Representative gonadal white adipose tissue (gWAT) histological images (H&E stain) scale bars 50 µM (**A**), calculated adipocyte volume (**B**) and adipocyte number (**C**) of female chow (**C**)- or high-fat (HF)-fed 30-week-old offspring of dams fed a chow (**C**) or HF diet for 6 weeks prior to pregnancy and during pregnancy and lactation. *N* = 5. * *p* < 0.05, ** *p* < 0.01.

To assess whether gWAT expansion in response to maternal obesity reflects hypertrophy, hyperplasia, or both, we conducted histological analysis of gWAT of 30-week-old HF- and chow-fed female offspring of lean and obese dams (Figure 2A). As expected, HF-fed offspring regardless of maternal diet had increased adipocyte volume, consistent with adipocyte hypertrophy (Figure 2A). Interestingly, we observed chow-fed offspring of obese dams exhibited increased adipocyte volume compared to those of lean dams (HF/C vs. C/C) (Figure 2A), suggesting that maternal obesity led to adipocyte hypertrophy in offspring, even if they were fed a chow diet throughout adulthood (Figure 2A,B). 

There were no differences in adipocyte volume between HF/HF and C/HF offspring (Figure 2A,B), indicating that maternal obesity followed by post-weaning HF feeding in offspring does not induce hypertrophy beyond what would be expected with post-weaning HF feeding. This suggests that the expansion of gWAT in HF/HF offspring compared to C/HF offspring may be explained by adipocyte hyperplasia (Figure 2B). Indeed we observed that while there was a significant increase in adipocyte number in HF-fed offspring of lean dams compared with that of chow-fed offspring of lean dams, there was a considerably greater rise in adipocyte number in HF-fed offspring of obese dams compared with HF-fed offspring of lean dams (HF/HF vs. C/HF), suggesting that hyperplasia is predominantly responsible for the enhanced gWAT expansion in HF/HF offspring (Figure 2C). 

### 3.3. Maternal Obesity Does Not Affect Developmental Adipogenesis

**Figure 3:**
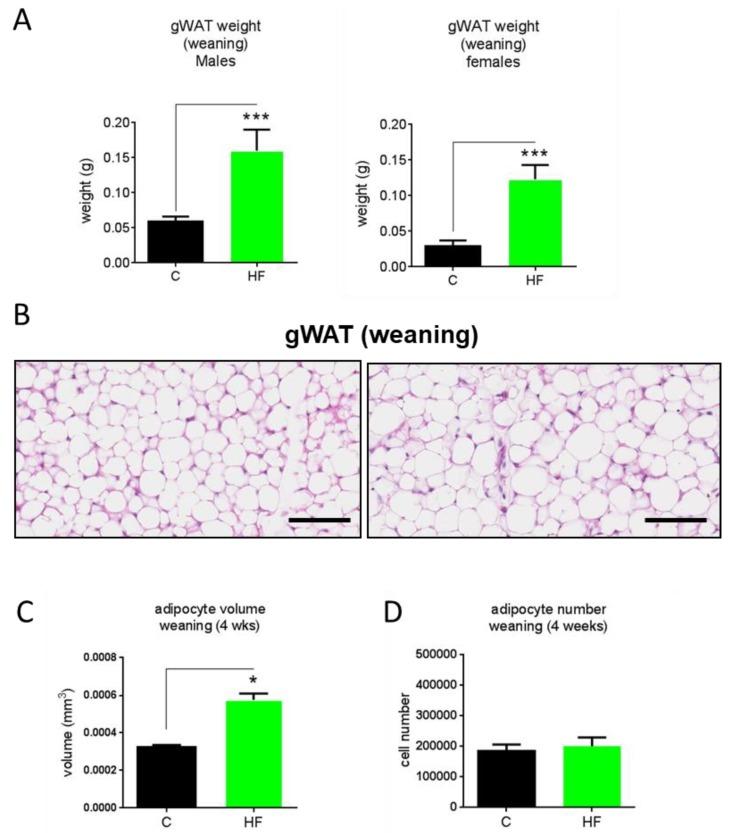
Gonadal white adipose tissue weights at weaning (4 weeks old) in male and female offspring (**A**), representative gWAT histological images (H&E stain), scale bars 50 µM (**B**), calculated adipocyte volume (**C**) and number (**D**) of dams fed a chow (**C**) or high-fat (HF) diet for 6 weeks prior to pregnancy and during pregnancy and lactation. *N* = 5. * *p* < 0.05, *** *p* < 0.001.

To assess whether maternal obesity affects developmental adipogenesis, which is complete by 4 weeks of age [21], we conducted a histological analysis of the gWAT of 4-week-old male and female offspring of lean and obese dams (Figure 3A). We observed that 4-week-old offspring of obese dams had increased gWAT weights (Figure 3A). Morphological examination revealed that the 4-week-old offspring of obese dams had increased adipocyte size (Figure 3B). Histological analysis revealed that adipocyte volume was increased in 4-week-old offspring of obese dams compared to 4-week-old offspring of lean dams (Figure 3C). Interestingly, there was no difference in adipocyte number between 4-week-old offspring of obese dams and those of lean dams, suggesting that maternal obesity has no effect on developmental adipogenesis (Figure 3D). 

### 3.4. Maternal Obesity Modifies Expression of Genes Involved in Adipocyte Progenitor Proliferation and Differentiation

**Figure 4:**
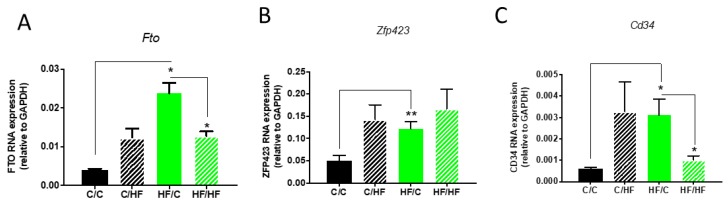
*Fto* (**A**), *Zfp423* (**B**), and *Cd34* (**C**) RNA expression in gWAT of female chow (C)- or high-fat (HF)-fed 30-week old offspring of dams fed a chow C or HF diet for 6 weeks prior to pregnancy and during pregnancy and lactation. All data represent means ± SEM. Data was analysed by ANOVA and Tukey’s multiple comparison test. *N* = 5–7. * *p* < 0.05, ** *p* < 0.01.

We next assessed the expression of markers of adipocyte progenitor proliferation/differentiation in the gWAT of 30-week-old female offspring. We observed that *Fto* gene expression was elevated in chow-fed offspring of obese dams compared to chow-fed offspring of lean dams. Interestingly, *Fto* expression was reduced in HF/HF offspring compared with HF/C offspring. *Zfp423* gene expression was elevated in HF/C vs. C/C offspring, but expression levels were not significantly different compared to HF/HF offspring (Figure 4). We observed that *Cd34* gene expression was elevated in HF/C offspring compared with C/C offspring. Interestingly, *Cd34* expression was significantly reduced in HF/HF offspring compared to HF/C offspring. 

## 4. Discussion

Our results are in agreement with numerous animal studies which have unequivocally shown that maternal obesity promotes obesity in adult offspring [6]. Furthermore, the results are, to the best of our knowledge, the first to investigate the effects of long-term (>16 weeks) high-fat feeding in mouse offspring of obese dams. We observed that maternal obesity in pregnancy and lactation leads to increased body weight and enhanced adiposity in response to a post-weaning HF challenge in offspring post-weaning compared to HF-fed offspring of lean dams. These results suggest that feeding offspring a HF diet exacerbates the obesity susceptibility triggered by maternal obesity, as there is a clear interaction between maternal and offspring diets. Interestingly, we observed that at weaning (4 weeks of age) the gWAT of offspring from obese dams exhibited increased adipocyte volume but no changes in adipocyte number, which indicates that maternal obesity during pregnancy and lactation promotes adipocyte hypertrophy but does not impact on developmental adipogenesis. This hypertrophic response of offspring adipocytes is in line with several reports in sheep [23,24], which documented that maternal obesity during pregnancy induced adipocyte hypertrophy during the neonatal period through enhanced lipid biosynthesis pathways and increased glucose uptake into the adipocytes. Our findings suggest that adipocyte hypertrophy and not hyperplasia is responsible for the increased gWAT weights at weaning of offspring of obese dams. Interestingly, we observed no significant difference in total gWAT adipocyte number between weanling offspring (irrespective of maternal diet) and those fed chow diet for 26 weeks post-weaning. This suggests that by the time of weaning, organogenesis of the gWAT is fully complete and that adipocyte number is not increased further for the duration of adulthood under chow conditions, which is in agreement with a recent study by Wang et al. [21]. Adipocyte volume of gWAT, on the other hand, was increased in 30-week-old chow-fed offspring of lean dams compared to weanling offspring of lean dams. This observation indicates that even under normal conditions, a considerable degree of adipocyte hypertrophy occurs after organogenesis in mice, and may represent the major means by which gWAT expands in adulthood under chow conditions. Interestingly, 30-week-old chow-fed offspring of obese dams exhibited adipocyte hypertrophy as compared to 30-week-old chow-fed offspring of lean dams, suggesting that even under chow post-weaning nutrition, maternal obesity may promote increased lipid deposition and storage within adipocytes in offspring.

Our findings that maternal obesity during pregnancy and lactation had no impact on developmental adipogenesis of the gWAT appear to contradict the findings of a recent study by Liang et al. in mice using a similar nutritional paradigm [25]. The study showed that maternal obesity in pregnancy and lactation led to an increase in adipocyte number at weaning. The reason for this discrepancy is likely to reflect differences in weaning age between the two studies. In our study, animals were weaned at 28 days, while in the study by Liang et al. animals were weaned at 21 days [25]. In a recent elegant study by Wang and colleagues which set out to establish in vivo adipogenesis events throughout the life course in mice, it was shown that developmental adipogenesis of the gWAT occurs predominantly in early postnatal life, during the lactation period [21]. Importantly, Wang et al. showed that while the gWAT is close to being fully formed by 28 days, a considerable amount of adipocytes are formed after 20 days postnatally [21]. Therefore, developmental adipogenesis is not yet complete at the time point in which Liang et al. [25] assessed developmental adipogenesis. It is therefore possible that the differences in developmental adipogenesis observed by Liang et al. at 21 days would not be observed by 28 days, which suggest that while maternal obesity initially increases the rate of developmental adipogenesis, it does not affect overall adipocyte number of the depot by the time gWAT organogenesis is complete at around 28 days [25]. Further studies are required to address this question more directly.

Obesogenic adipogenesis is the major means by which gWAT expands in response to HF diet. Our results show that obesogenic adipogenesis, unlike developmental adipogenesis, may be programmed by maternal obesity. Offspring of lean dams fed a HF diet for 26 weeks post-weaning exhibited increased gWAT adipocyte number, which is as expected under such a dietary regimen known to induce obesogenic adipogenesis in mice [21]. Offspring of obese dams, however, fed a post-weaning HF diet for 26 weeks exhibited increased adipocyte number compared to HF-fed offspring of lean dams, clearly indicating that obesogenic adipogenesis is enhanced in the offspring of obese dams. A recent study, however, has documented a contradictory finding, suggesting that gWAT expansion capacity in offspring of obese dams is in fact reduced [25]. Liang et al. postulated that adulthood obesogenic adipogenesis in offspring of obese dams could be blunted due to their finding of increased developmental adipogenesis which may have reached full capacity [25]. However, this study, which used histological assessment of adipocyte volume and calculations of adipocyte number, employed a 9-week post-weaning HF diet. Obesogenic adipogenesis in response to HF diet has been shown to be still in progress beyond 12 weeks of HF-diet feeding in rodents, with a considerable amount of adipocytes that contribute to gWAT expansion yet to form by week 10 [21]. Thus, a 9-week HF diet regimen is insufficient time to allow for changes in gWAT hyperplasia to occur. Moreover, a recent study by Borengasser and colleagues [18], which set out to establish the adipogenic potential of gWAT of adult rat offspring of obese dams, found that SFV cells of the gWAT of the offspring of obese dams have increased adipogenic capacity ex vivo at both 21 days and 3 months postnatal [18]. The latter time point is the same as that used by Liang et al. to assess adipocyte number in mice. Therefore, contrary to the findings of Liang et al., our results are in agreement with those of Borengasser et al., which demonstrate enhanced adipogenic capacity in adult offspring of obese dams. While we postulate that enhanced obesogenic adipogenic capacity in offspring of obese dams represents developmental programming of adipocyte progenitors induced by maternal obesity, we cannot rule out that this effect is due to the gWAT reaching maximal triacylglycerol storage capacity. Our results showing that maternal obesity induces offspring adipocyte hypertrophy, even under chow conditions, may support this notion. 

Previous studies have failed to fully establish whether offspring developmental or obesogenic adipogenesis, or perhaps both, are affected by maternal obesity. Our results indicate that maternal obesity promotes adulthood obesogenic adipogenesis in the gWAT of offspring. Emerging evidence is beginning to establish the fact that, rather than representing different time-dependent versions of the same process, developmental and adulthood adipogenic events are regulated by distinct signalling pathways and transcriptional events and require distinct subpopulations of APs [14,15,16]. We observed that mRNA expression levels of AP markers *Zfp423* and *Cd34* were elevated in the gWAT of chow-fed offspring of obese dams at 30 weeks of age. However, *Cd34* expression was reduced in the 30-week-old HF-fed offspring of obese dams compared to chow-fed offspring of obese dams. Given that *Cd34*-positive APs become negative for *Cd34* as they differentiate into mature adipocytes [8], these data may support the notion that maternal obesity leads to an increased proliferation and presence of APs in the stromal vascular fraction of offspring gWAT, which, when offspring are exposed to a post-weaning HF diet, contribute to the enhanced adipogenic response to obesogenic nutritional stimuli in adulthood. Work from our group has previously established that, while playing no role in developmental adipogenesis, the *Fto* gene plays a critical role in obesogenic adipogenesis of the gWAT in adult mice by promoting AP proliferation [22]. In the present study, we found that *Fto* expression was increased in the gWAT of 30-week-old chow-fed offspring of obese dams compared to chow-fed offspring of lean dams, while it was reduced in the gWAT of HF-fed offspring of obese dams compared to chow-fed offspring of obese dams. 

Taken together, these results support the notion that maternal obesity promotes the proliferation of APs in offspring gWAT that reside in the vascular niche, and although developmental adipogenesis is not affected, there is a considerable impact on obesogenic adipogenesis in adult offspring. A recent study has determined that a distinct and dynamic AP proliferation phase is required for obesogenic adipogenesis [14]. This study documented that just 3 days of HF-diet feeding in mice is sufficient to promote a rapid AP proliferation phase followed by a latent period of several weeks before hyperplasia occurs. The mechanistic basis behind this rapid proliferation phase and latent period before this results in adipogenesis is unknown. Previous studies assessing ex vivo adipogenic capacity of offspring gWAT in response to maternal obesity have established that epigenetic alterations in *Zfp423* are required for the enhanced adipogenic capacity, though increased AP proliferation must by upstream of this event, as *Zfp423*, while important for adipocyte cell fate determination and commitment, has never been linked to AP proliferation [26]. Based on our findings and those of others, we postulate that *Fto* is a key mediator of the enhanced obesogenic adipogenesis in the offspring of obese dams through its effects on AP proliferation. This may be an important mechanism by which maternal obesity promotes obesity in offspring. 

Changes in adipogenic capacity per se are unlikely to completely account for changes in adiposity, and therefore differences in energy expenditure (alterations in metabolic rate and/or physical activity) or food intake may cause or exacerbate increased fat deposition in offspring of obese dams. Future studies that directly assess food intake and energy expenditure in the offspring of obese dams is required to confirm this. A potential limitation of the study is that histological and gene expression analysis was only conducted in female offspring and so sex differences in adipogenic response cannot be ruled out. Certainly, female offspring of obese dams exhibit more statistically significant increases in adiposity than males, compared to their respective controls. 

## 5. Conclusions

In conclusion, we have revealed that maternal obesity has long-lasting effects on the hyperplastic and hypertrophic response in offspring visceral adipose tissue. Our results suggest that the hyperplastic response of visceral adipose tissue may be programmed during development by maternal obesity. The metabolic consequences of the enhanced hyperplastic response of offspring adipose tissue induced by maternal obesity is yet to be determined, but may represent a compensatory response to overnutrition that is geared towards more efficient and metabolically safe energy storage

## Figures and Tables

**Table 1:** Energy content of the diets.

% Kcal From:	C	HF
Fat	7.4	45
Protein	17.5	20
Carbohydrate	75.1	35
kcal per gram	3.53	4.54

C: Chow; HF: high-fat diet.
